# Activation of RidA chaperone function by *N*-chlorination

**DOI:** 10.1038/ncomms6804

**Published:** 2014-12-17

**Authors:** Alexandra Müller, Sina Langklotz, Nataliya Lupilova, Katja Kuhlmann, Julia Elisabeth Bandow, Lars Ingo Ole Leichert

**Affiliations:** 1 Institute of Biochemistry and Pathobiochemistry—Microbial Biochemistry, Ruhr-Universität Bochum, Universitätsstrasse 150, 44780 Bochum, Germany; 2 Biology of Microorganisms, Ruhr-Universität Bochum, Universitätsstrasse 150, 44780 Bochum, Germany; 3 Medizinisches Proteom-Center, Ruhr-Universität Bochum, Universitätsstrasse 150, 44780 Bochum, Germany

## Abstract

*Escherichia coli* RidA is a member of a structurally conserved, yet functionally highly diverse protein family involved in translation inhibition (human), Hsp90-like chaperone activity (fruit fly) and enamine/imine deamination (*Salmonella enterica*). Here, we show that *E. coli* RidA modified with HOCl acts as a highly effective chaperone. Although activation of RidA is reversed by treatment with DTT, ascorbic acid, the thioredoxin system and glutathione, it is independent of cysteine modification. Instead, treatment with HOCl or chloramines decreases the amino group content of RidA by reversibly *N*-chlorinating positively charged residues. *N*-chlorination increases hydrophobicity of RidA and promotes binding to a wide spectrum of unfolded cytosolic proteins. Deletion of *ridA* results in an HOCl-sensitive phenotype. HOCl-mediated *N*-chlorination thus is a cysteine-independent post-translational modification that reversibly turns RidA into an effective chaperone holdase, which plays a crucial role in the protection of cytosolic proteins during oxidative stress.

## Abstract (web summary)

Hypochlorous acid generated by neutrophils acts as a potent antibacterial agent. Müller *et al*. now show that this oxidant directly activates a protective counter-response in *E. coli* by *N*-chlorinating the protein RidA and converting it into an effective protein chaperone.

## Graphical abstract

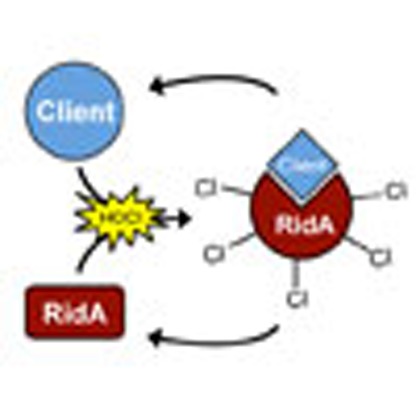

Hypochlorous acid (HOCl) is not only a very potent disinfectant, but functions as a physiological oxidant released by neutrophils to fight infections (for a comprehensive overview see ref. 1). Once activated, neutrophils phagocytize pathogens and internalize them into the phagosome. During the oxidative burst, neutrophils then generate large amounts of superoxide anions (O_2_^·−^) and hydrogen peroxide (H_2_O_2_) at the expense of oxygen by NADPH-oxidases. Myeloperoxidase converts this H_2_O_2_ and chloride ions into HOCl, which together with the other products of the respiratory burst, is highly effective in killing microorganisms.

The mode of action of HOCl-dependent microbial killing involves reactions with a variety of biological molecules, including DNA, lipids, NADH, cholesterol, proteins and non-protein thiols such as glutathione^1^. Due to their high cellular abundance, however, proteins are considered to be the major targets of HOCl-mediated modifications. Damage caused by HOCl includes protein fragmentation, dimerization and side chain modification, typically leading to protein inactivation and degradation by the cellular protein quality control^2^,^3^,^4^. Methionine and cysteine residues react most rapidly with HOCl, while its reactivity with side chains of histidine, tryptophan, lysine, tyrosine and arginine is two to five orders of magnitude lower^4^.

As HOCl does not only target microbial proteins, but readily halogenates organic and inorganic primary amines present in the phagosome, chloramines such as monochloramine (NH_2_Cl) and *N*-chlorotaurine accumulate there as well^4^,^5^. Although being less reactive and more stable than HOCl, chloramines share the oxidizing and chlorinating properties of HOCl, making them equally useful as disinfectants in drinking water supplies, medicine and household settings^6^.

Pathogens have developed numerous strategies to successfully escape the immune response during infection (see ref. 7 for a comprehensive overview). Yet, HOCl-response mechanisms in bacteria remain largely elusive. In Firmicutes, such as *Bacillus subtilis* and *Staphylococcus carnosus*, inactivation of conserved metabolic enzymes and essential proteins such as methionine synthase and the redox-sensing repressor OhrR by S-bacillithiolation mediates the HOCl stress response^8^,^9^. *Salmonella enterica* specifically reduces the influx of HOCl through ArcA-dependent downregulation of the outer membrane porine OmpW in response to hypochlorite stress^10^. *Escherichia coli* responds to hypochlorite stress using a number of complementary strategies. Hydrogen peroxide and heat-shock response genes were shown to be regulated in response to hypochlorite stress^11^. The *E. coli* transcription factors NemR, HypT and RclR are involved in hypochlorite tolerance by the regulation of genes involved in electrophile detoxification, sulfur metabolism and iron acquisition, and survival upon chlorine stress, respectively^12^,^13^,^14^,^15^. Furthermore, the redox-regulated chaperone Hsp33 is activated by oxidative unfolding mediated by HOCl. The activated state of Hsp33 protects cellular proteins from HOCl-induced aggregation by direct protein–protein interaction under conditions that prevent ATP-dependent chaperones from working properly^16^.

In this study, we describe a novel mechanism, which confers protection of *E. coli* against HOCl stress-mediated protein aggregation. This mechanism is dependent on RidA, a member of the functionally highly diverse YjgF/YER057c/UK114 protein family. Although highly conserved across kingdoms, members from this protein family display very different functions. They are, for instance, involved in translation inhibition (rat), activation of calpain protease (cow), adenine-mediated repression of purine biosynthesis (*Bacillus subtilis*) and mitochondrial maintenance (*Saccharomyces cerevisiae*)^17^,^18^,^19^,^20^. The *Salmonella enterica* homologue RidA has recently been described as an enamine/imine deaminase, playing a crucial role in the synthesis of branched-chain amino acids^21^.

Here, we show that *E. coli* RidA acts additionally as redox-regulated chaperone that is activated on exposure to HOCl stress. Although many proteins are inactivated on reaction with HOCl and precipitate, RidA turns into a potent chaperone, preventing highly abundant cellular proteins from HOCl-induced aggregation. Surprisingly, unlike in the other known redox-regulated chaperone Hsp33, where activation involves reversible disulfide bond formation^22^, activation of RidA’s chaperone activity is mediated by *N*-chlorination, a modification that has previously been thought to be deleterious to protein function. We also observe activation of chaperone function via *N*-chlorination in the *Drosophila melanogaster* RidA homologue DUK114, as well as in a few unrelated proteins, indicating that this is a novel post-translational modification that induces affinity for aggregation-sensitive folding intermediates. In addition to reduction by glutathione, thioredoxin is capable of reducing *N*-chlorinated RidA, a hitherto unknown function of this well-characterized thiol-disulfide oxidoreductase.

## Results

### HOCl-treated RidA inhibits threonine dehydratase activity

**Figure 1:**
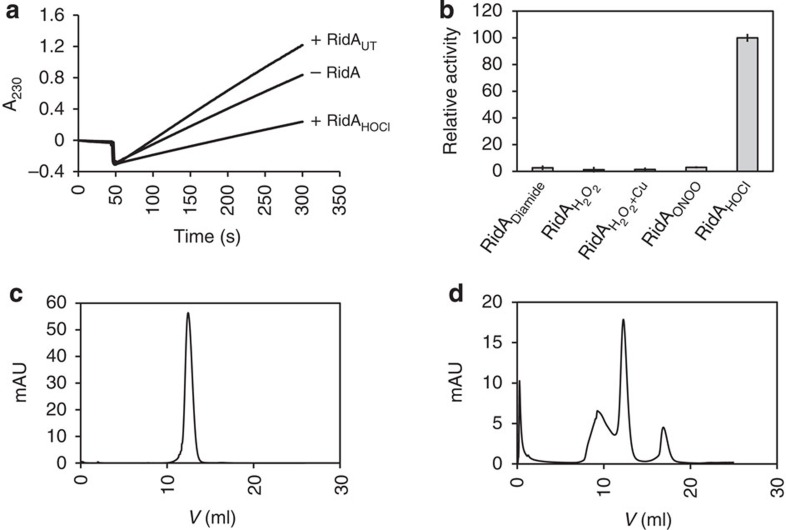
HOCl-treated RidA inhibits IlvA. (**a**) Untreated RidA (RidA-UT) accelerates α-ketobutyrate generation from threonine catalysed by threonine deaminase IlvA. In the presence of RidA treated with a 10-fold excess of HOCl for 5 min at 37 °C, product formation is clearly decreased below levels of product formation in the absence of RidA indicating inhibition of IlvA. A representative measurement is shown. (**b**) RidA was incubated with a 10-fold molar excess of diamide, peroxynitrite (ONOO^−^) or HOCl, or a 5-fold molar excess of H_2_O_2_ or H_2_O_2_/CuCl_2_ for 10 min at 30 °C. Oxidants were removed before analysis of chaperone activity. Relative chaperone activities were calculated by normalizing the change of absorption to HOCl-treated RidA. Diamide, H_2_O_2_ and peroxynitrite (ONOO^−^) do not induce chaperone activity of RidA. The oligomeric state of RidA without prior treatment (**c**) or after treatment with a 10-fold excess of HOCl for 5 min at 37 °C (**d**) was analysed. HOCl was removed with the help of Micro Bio-Spin Chromatography Columns before gel filtration using a Superdex 75 10/300 GL column.

*Salmonella enterica* RidA has previously been reported to act as deaminase of reactive enamine/imine intermediates, stimulating IlvA-mediated degradation of threonine to 2-ketobutyrate^21^. We recently discovered that modification of a single cysteine residue in RidA by peroxynitrite treatment inhibits this stimulatory function^23^. We tested the effect of several other nitrosative and oxidative stressors on RidA’s enamine/imine deaminase activity. Intriguingly, when treated with the physiological disinfectant sodium hypochlorite (HOCl), RidA not only lost its stimulatory effect in the threonine deaminase assay, but inhibited the overall rate of the reaction to levels below the ones observed in the absence of RidA (Fig. 1a). These results could no longer be explained by a simple inhibition of RidA alone but suggested that the threonine dehydratase IlvA is directly inhibited by HOCl-treated RidA (RidA_HOCl_), potentially through a tight direct protein–protein interaction of the two proteins. We, therefore, hypothesized that RidA, once treated with HOCl, might be a chaperone that effectively binds other proteins.

### RidA is a chaperone activated by HOCl

**Figure 2:**
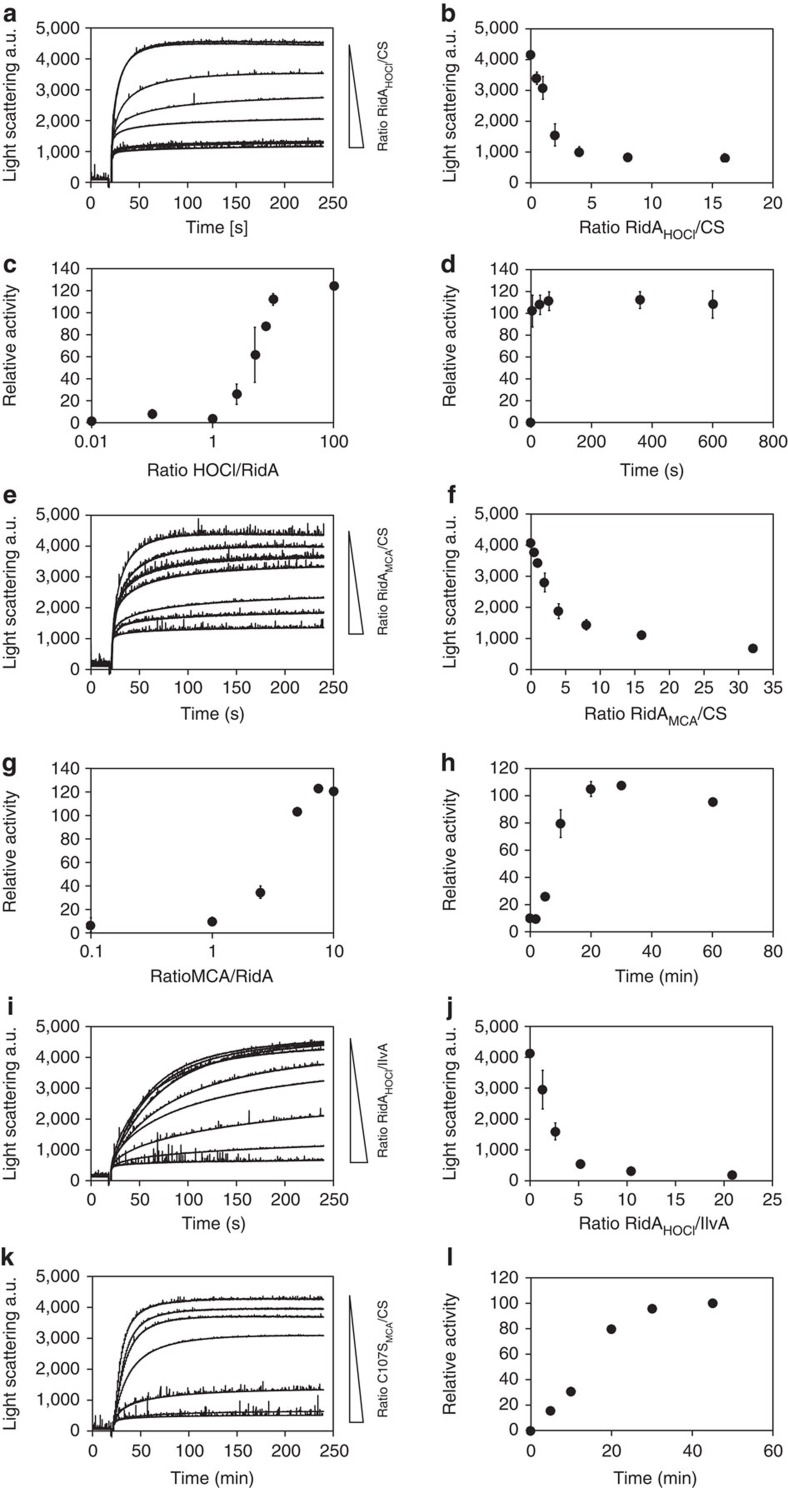
RidA is chaperone activated by HOCl and monochloramine. (**a**) HOCl-treated RidA inhibits aggregation of chemically denatured citrate synthase in a concentration-dependent manner as measured by light scattering at 360 nm. Ratios of RidA_HOCl_ over citrate synthase are indicated in **b**. (**b**) Inhibition of light scattering is linear at ratios of RidA over citrate synthase between 0.5 to 5 and reaches a maximum at higher ratios. (**c**) Activation depends on the ratio of HOCl over RidA. Activation reaches a maximal value at a 10-fold excess of HOCl. (**d**) Activation of RidA by HOCl occurs already within mixing time. (**e**,**f**) Chaperone activity of RidA is activated by monochloramine. (**g**) Activation by monochloramine requires similar ratios of monochloramine over RidA as shown for HOCl-dependent activation. (**h**) Activation of RidA by monochloramine is significantly slower when compared with activation by HOCl. (**i**,**j**) HOCl-treated RidA inhibits IlvA aggregation by direct binding. (**k**,**l**) Activation of the cysteine-free variant RidA-C107S by MCA occurs in a similar time range as shown for WT-RidA. Calculation of relative activities is represented as mean of three independent measurements in **b** or two independent measurements in **c**,**d**,**f**,**g**,**h**,**j**,**l**. A representative measurement is shown for **a**,**e**,**i**,**k**.

**Figure 3:**
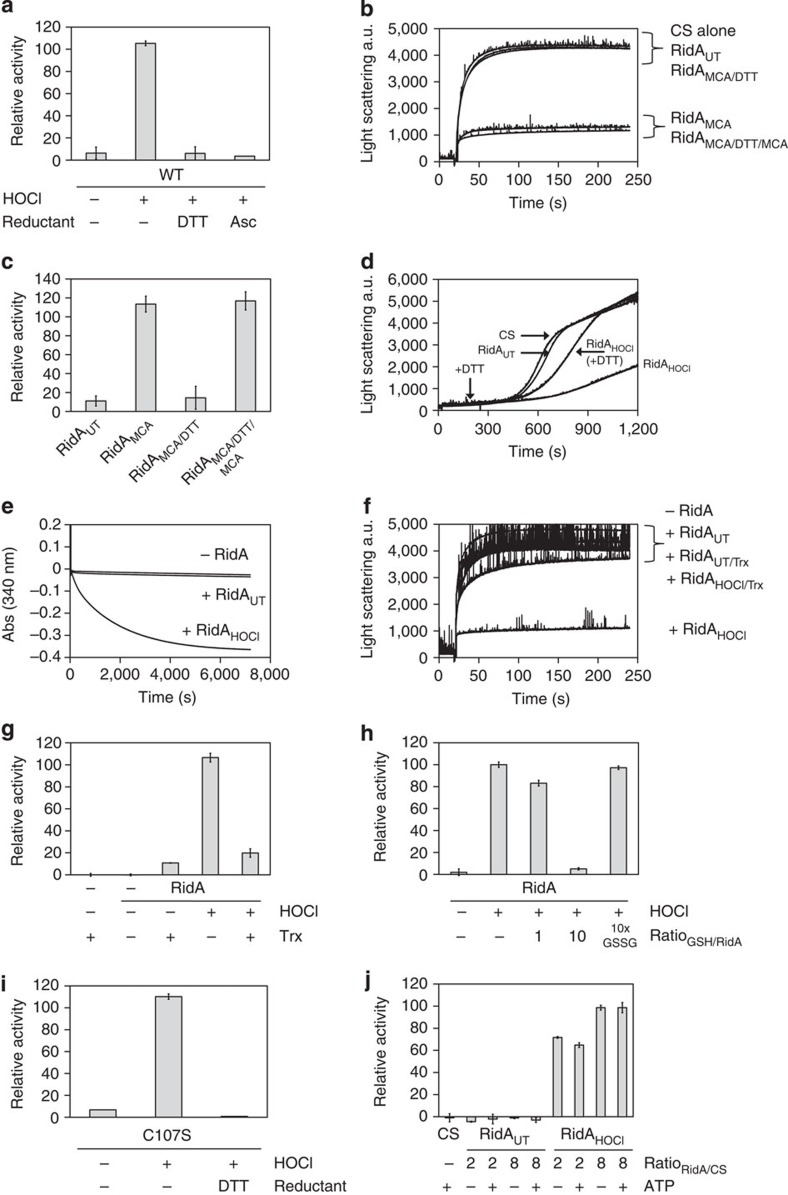
RidA activation by HOCl is reversible and does not depend on cysteine modification. (**a**) Treatment of RidA_HOCl_ with DTT or ascorbic acid (Asc) leads to inactivation. (**b**,**c**) After inactivation of RidA_MCA_ with DTT, the protein can be reactivated with monochloramine. (**d**) Aggregation of heat-denatured citrate synthase was analysed in the absense or the presence of untreated or HOCl-treated RidA. DTT was added after 250 s to the RidA_HOCl_-containing cuvette (arrow) to monitor release of citrate synthase upon reduction. (**e**) Thioredoxin exclusively reduces RidA_HOCl_ as measured by the consumption of NADPH. (**f**,**g**) Samples from **e** were subjected to a citrate synthase aggregation assay. Activity of thioredoxin-reduced RidA is strongly diminished. (**h**) RidA_HOCl_ chaperone activity is abolished after treatment with a 10-fold molar excess of reduced glutathione (GSH). Treatment with oxidized glutathione (GSSG) does not alter activity. (**i**) A cysteine-free variant of RidA is still as active as wild-type RidA and activation is likewise reversible. (**j**) Chaperone holdase activity of RidA_HOCl_ is independent of ATP. Addition of ATP to untreated RidA does not induce activation. Data are represented as means and standard deviations from three independent measurements in **a** and **c** and two independent measurements in **g**,**h**,**i** and **j**. Representative measurements for **b**,**d**,**e** and **f** are shown.

To test RidA_HOCl_ for chaperone activity, we performed aggregation assays using chemically denatured citrate synthase and untreated and HOCl-treated RidA. RidA was incubated with a 10-fold molar excess of HOCl, a concentration that is sufficient to fully activate the well-described HOCl-activated chaperone Hsp33 (ref. 16). Aggregation of chemically denatured citrate synthase was prevented by RidA_HOCl_ in a dose-dependent manner (Fig. 2a,b). Other oxidants including H_2_O_2_ or diamide did not activate RidA’s chaperone activity, suggesting that the activation was indeed HOCl-specific (Fig. 1b). A fivefold excess of RidA_HOCl_ was sufficient to completely inhibit citrate synthase aggregation, suggesting that HOCl is a potent activator of RidA chaperone activity (Fig. 2b). These concentration ratios are similar to other chaperones, such as Hsp33. Analysis of the activation kinetics revealed that a 10:1 molar ratio of HOCl over RidA was sufficient for full chaperone activity within the first 5 s of incubation (Fig. 2c,d). The addition of ATP did not enhance chaperone activity, consistent with a holdase function of HOCl-treated RidA (Fig. 3j).

To determine the impact of HOCl treatment of RidA on its oligomeric state of RidA, we performed analytical gel filtration (Fig. 1c,d). For this purpose, untreated RidA and RidA_HOCl_ were loaded on a Superdex 75 10/300 GL column to monitor the molecular weight of the protein complex. While untreated RidA eluted as a trimer as reported previously^23^ (Fig. 1c), treatment of RidA with HOCl partially coincides with the formation of higher molecular weight oligomers (Fig. 1d). This points towards a tendency of RidA to homo-oligomerize when treated with HOCl. Oligomerization thus may be a requirement for binding of unfolding proteins under these conditions.

### RidA is activated by monochloramine

Within the phagosome, most of the produced HOCl readily reacts with free ammonia or amino acids resulting in the formation of chloramines such as monochloramine or *N*-chlorotaurine^1^. In fact, HOCl-derived chloramines are discussed to be the major contributors for the antimicrobial activity of HOCl^24^,^25^. Thus, we investigated whether monochloramine (NH_2_Cl; MCA) is also capable of activating the chaperone function of RidA. Indeed, monochloramine-treated RidA (RidA_MCA_) also prevented citrate synthase aggregation (Fig. 2e,f). RidA activation was maximal with a 10:1 molar ratio of MCA over RidA (Fig. 2g). In contrast to HOCl, however, RidA activation by MCA proceeded at much lower rates and required at least 20 min incubation to achieve full chaperone activity (Fig. 2h). This finding may be explained by the lower absolute rate constants of the reaction of chloramines with biomolecules when compared with HOCl^26^,^27^.

### Unfolded IlvA is a direct substrate of RidA

To assess whether RidA_HOCl_ is capable of also interacting with IlvA, we established aggregation assays substituting citrate synthase with IlvA. We found that chemically denatured IlvA readily aggregates upon dilution into assay buffer (Fig. 2i). RidA_HOCl_ prevented aggregation of IlvA in a concentration-dependent manner (Fig. 2i,j). In contrast, incubation of IlvA with a 10-fold molar excess of untreated RidA had no effect on IlvA’s aggregation behaviour (Fig. 2i). IlvA thus is a bacterial protein suitable to be used as a substrate in chaperone assays.

### RidA activation is reversible by reduction

To test whether the activation of RidA’s chaperone activity is a reversible process, we tested chaperone activity of RidA_MCA_ after treatment with the thiol-based reductant dithiothreitol (DTT). Indeed, DTT-reduced RidA_HOCl_ and RidA_MCA_ were no longer able to prevent citrate synthase aggregation (Fig. 3a–c). To analyse if the reduced RidA_MCA_ can be activated again, we did another series of citrate synthase aggregation assays (Fig. 3b,c). RidA activity could be recovered by subsequent treatment with MCA (Fig. 3b,c). This demonstrates that modification by MCA followed by reduction with DTT does not compromise integrity and functionality of the protein. RidA thus is a novel redox-regulated protein activated by HOCl and chloramines.

We also performed thermal aggregation assays at 43 °C using citrate synthase to analyse whether reduction of activated RidA results in substrate release (Fig. 3d). Citrate synthase did aggregate significantly slower in the presence of RidA_HOCl_. However, when DTT was added to the reaction mixture after 250 s, citrate synthase started to aggregate, indicating that reduction of RidA_HOCl_ is accompanied by the release of its substrate.

### RidA activation is reversible by thioredoxin and glutathione

In bacteria, the reductive redox state of cytosolic proteins is maintained by thioredoxins and glutaredoxins^28^,^29^. Both proteins have been shown to reduce disulfide-containing substrate proteins by direct thiol-disulfide exchange reactions. The disulfide bond, which is subsequently formed in thioredoxin and glutaredoxin is then reduced by the NADPH-dependent thioredoxin reductase or glutathione, respectively^28^. In *E. coli*, the thioredoxin system is composed of thioredoxin A (TrxA) and thioredoxin reductase (TrxB).

To test whether *E. coli* TrxA is involved in the inactivation of RidA, we reconstituted the complete thioredoxin system comprising TrxA, TrxB and NADPH *in vitro*. Activity of TrxA was monitored by the consumption of NADPH (measured at 340 nm). In the absence of RidA_HOCl_, we did not observe any significant decrease in absorption (Fig. 3e). In contrast, however, addition of 50 μM of RidA_HOCl_ to the reaction mixture resulted in the significant consumption of NADPH. Moreover, thioredoxin-dependent reduction of RidA caused a loss of chaperone activity as observed in citrate synthase aggregation assays (Fig. 3f,g). The overall velocity of thioredoxin-dependent reduction of RidA_HOCl_ observed could be substantially different from a reduction velocity *in vivo*, as the amount of NADPH used in our experimental setup limits the reduction rates. To further elaborate if reduction by other thiol-based reducing systems present in the cell may occur, we incubated RidA_HOCl_ with excess glutathione (Fig. 3h). While oxidized glutathione (GSSG) did not have any impact on RidA_HOCl_ activity, the addition of reduced glutathione (GSH) blocked its holdase function.

### Loss of free amino groups by HOCl treatment

On the basis of our previous observation that RidA is oxidized at its single cysteine residue (C107) upon peroxynitrite treatment^23^, we assumed that HOCl-dependent activation of RidA is also mediated by modification of C107. Strikingly, however, we found that the cysteine-free variant RidA-C107S was still as active as the wild-type protein after HOCl treatment, and that activation of the treated mutant variant was reversible by DTT as well (Fig. 3i). This result strongly points towards a novel mechanism of functional activation by HOCl, independent of cysteine.

Apart from the oxidation of cysteine thiols, HOCl is also known to react with a number of other amino acid side chains, including methionine, histidine, lysine, arginine, tryptophan and tyrosine^4^. Reaction of HOCl with side chain amines of lysine, for instance, results in *N*-chlorination to mono- and dichloramines. To analyse whether HOCl-mediated activation of RidA involves the chlorination of side chain amines, RidA_HOCl_ was treated with ascorbic acid. In contrast to DTT, ascorbic acid does not significantly reduce cysteine oxidation products but readily reacts with chloramines allowing the discrimination of these modifications^30^,^31^. As shown in Fig. 3a, incubation of RidA_HOCl_ with ascorbic acid caused a complete loss of chaperone activity supporting the idea that RidA is a chaperone activated by *N*-chlorination.

**Figure 4:**
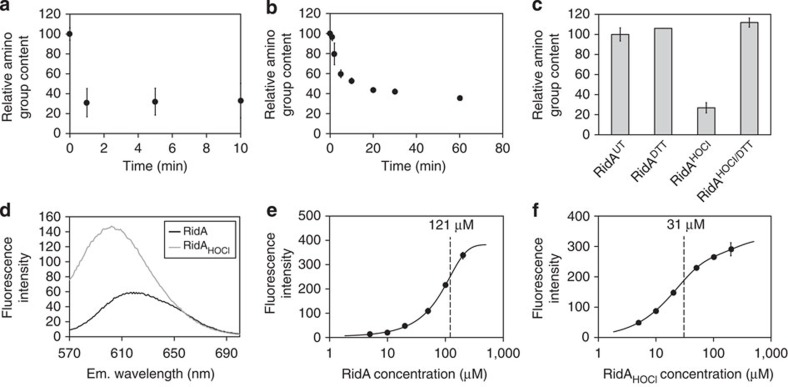
RidA activation is accompanied by a loss of free amino groups and an increase in hydrophobicity. (**a**) Amino group content of RidA was analysed after incubation with HOCl at indicated time points using fluorescamine. The decrease in amino group content drops to <40% at the first time point measured (1 min). (**b**) Monochloramine treatment of RidA results in a slower decrease of amino group content. (**c**) DTT treatment of RidA leads to full recovery of amino groups. (**d**) Fluorescence of 0.2 μM nile red was analysed in the presence of 50 μM RidA and 50 μM RidA_HOCl_. A wavelength and maximum shift is observable for RidA_HOCl_ when compared with RidA. The increase in absolute fluorescence of 0.2 μM nile red at different protein concentrations was determined at 613 nm for RidA (**e**) and at 600 nm for RidA_HOCl_ (**f**). The calculated half-saturation in **e** and **f** is displayed by a vertical line. Means and variances in **a**,**b**,**c**,**e** and **f** are based on two independent measurements. A representative measurement for **d** is shown.

*N*-chlorination of lysine and arginine side chains inevitably decreases the total amount of detectable amino groups. RidA possesses 14 potential *N*-chlorination targets: five arginine residues, eight lysine residues and one terminal amino group. We monitored the accessibility of these potential targets upon HOCl or monochloramine treatment using fluorescamine^32^. The amino group content of RidA decreased to <40% within seconds of HOCl treatment and was not further reduced upon longer treatment (Fig. 4a). Monochloramine treatment led to a much slower loss of amino groups but reached the same final percentage (Fig. 4b). Disappearance of amino groups, therefore, appeared to parallel the appearance of RidA’s chaperone function (compare Fig. 2d,h). The observed 60% reduction of amino group content corresponds to approximately eight residues that no longer contain a free amino group. DTT treatment leads to a full recovery of accessible amino groups, which coincides with the inactivation of RidA (compare Fig. 4c and Fig. 3a).

### HOCl treatment increases hydrophobicity of RidA

The above-described loss of positive charges prompted us to conclude that binding of substrate proteins may be enabled by an increase in hydrophobicity. We thus used the uncharged hydrophobic dye Nile red to directly monitor the polarity of RidA^33^. The fluorescent signal of 0.2 μM Nile red was compared after mixing with either 50 μM RidA or 50 μM RidA_HOCl_ (Fig. 4d). We observed both a wavelength shift from 613 to 600 nm and an increase in absolute fluorescence for RidA_HOCl_ when compared with untreated RidA (Fig. 4d). The RidA and RidA_HOCl_ concentrations leading to half-saturation of the proteins with the dye were determined (Fig. 4e,f). This concentration was significantly higher for untreated RidA (121.1 μM; Fig. 4e) when compared with RidA_HOCl_ (30.7 μM; Fig. 4f). These results indicate that RidA_HOCl_ has an increased hydrophobicity when compared with untreated RidA, which most likely enables binding of unfolding substrates to RidA_HOCl_.

### RidA is *N*-chlorinated by HOCl and monochloramine

**Figure 5:**
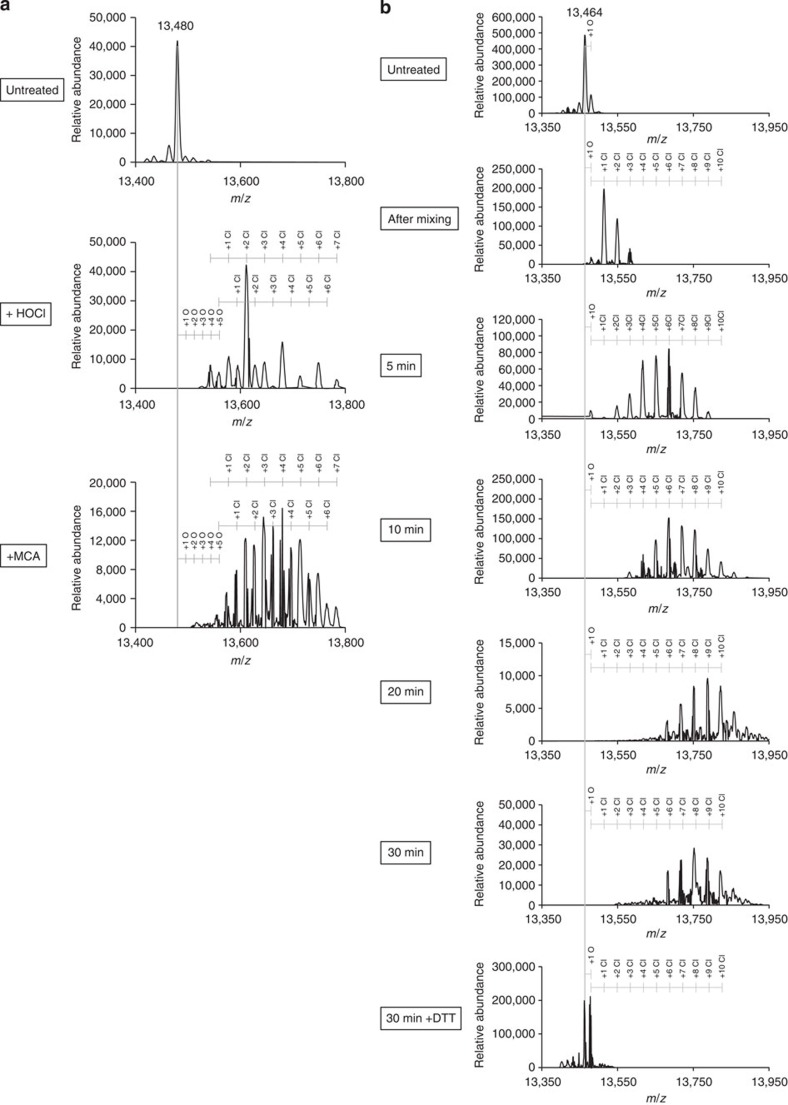
RidA activation is mediated by *N*-chlorination. (**a**) Full-length untreated, HOCl-treated and MCA-treated RidA was analysed in an OrbiTrap Elite mass spectrometer. Up to seven chlorine atoms (and five oxygen atoms) are added to RidA. (**b**) Time-resolved analysis of MCA-mediated modification of RidA-C107S. Samples for mass spectrometry were taken directly after addition of MCA and after time points indicated. After 30 min of incubation, DTT reduction was carried out for 30 min before mass spectrometry.

To identify the exact type of modification that activates RidA, we performed mass spectrometry of full-length RidA (Fig. 5a). After HOCl treatment, the peak at 13,480 Da corresponding to unmodified RidA fully vanished. Instead, a number of peaks corresponding to the addition of multiple (up to seven) chlorines appeared. In addition to chlorine adducts, we also observed the irreversible addition of up to five oxygen atoms to methionine and cysteine residues. These oxidation products seemed less prominent after monochloramine treatment of RidA. To exclude the oxidizing effects of chlorinating agents on cysteine, we used the cysteine-free RidA variant C107S for the analysis of a time-resolved monochloramine treatment (Fig. 5b). The chlorination of RidA-C107S observed in this experiment paralleled the activation of chaperone activity (compare Fig. 2k,l and Fig. 5b). The number of chlorines added to RidA-C107S consecutively increased with incubation time until ~20 min, when the maximum number of added chlorine atoms was observed. Reduction with DTT resulted in the complete removal of chlorine residues, while oxygen, presumably added to methionine, remained (Fig. 5b).

### A *ridA* mutant is sensitive towards HOCl *in vivo*

**Figure 6:**
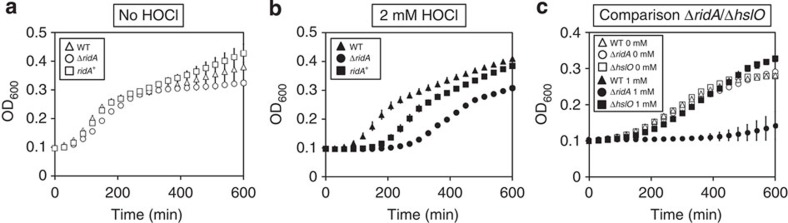
The lack of RidA leads to prolonged recovery from HOCl stress. Growth of *E. coli* BL21 wild type, a *ridA* mutant and a complementation strain overexpressing RidA in the presence of 2 mM HOCl (**b**) was compared with growth under control conditions (**a**). (**c**) Growth of a Hsp33 mutant (Δ*hslO;* AM02) was compared with the WT and the *ridA* mutant. For a more detailed overview of growth behaviours of all three strains at different HOCl concentrations, see Fig. 7. Means of OD_600_ values and standard deviations were calculated from three independent measurements.

**Figure 7:**
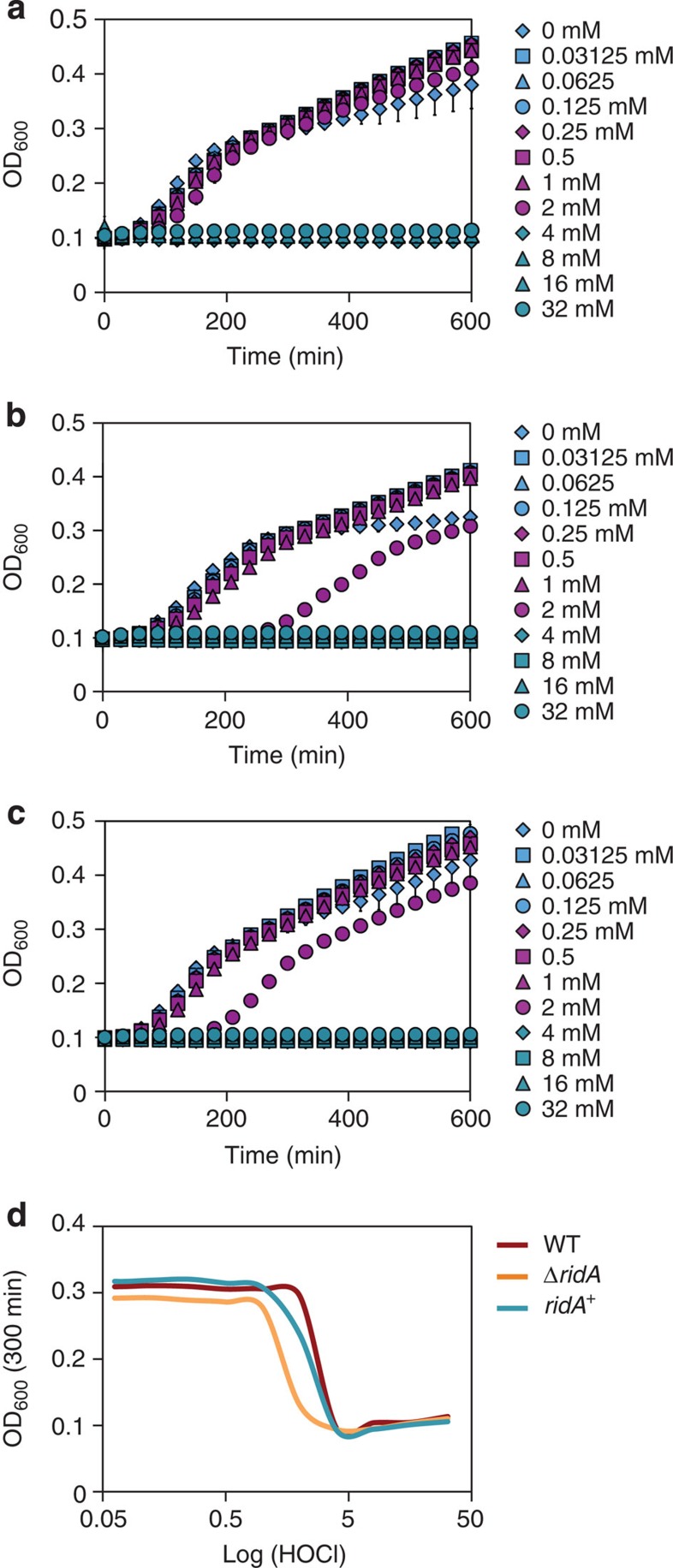
Dose-toxicity analysis of HOCl. The ability of *E. coli* BL21 WT (**a**), Δ*ridA* (**b**) and Δ*ridA* (*ridA*^+^) (**c**) to grow in the presence of HOCl (concentrations ranging from 0 to 32 mM) was analysed. A dose-toxicity curve based on final OD_600_ after 300 min of growth is provided for all the three strains (**d**). Means of OD_600_ values and standard deviations, as well as dose toxicity, were calculated from three independent measurements.

To address the question whether RidA protects cells against the consequences of HOCl-induced damage, we compared the growth of wild-type, *ridA* deletion mutant and a RidA overexpression strain in the absence and presence of HOCl (Figs 6 and 7). In the absence of HOCl, all the three strains showed a comparable growth behaviour (Fig. 6a). However, in the presence of medium HOCl concentrations (1–2 mM), the *ridA* mutant displayed a significantly prolonged lag phase when compared with the wild type (Figs 6b and 7b,d) and a Δ*hslO* mutant (deficient in the known HOCl-activated chaperone Hsp33; Fig. 6c). Overexpression of RidA in the *ridA* mutant resulted in an intermediate phenotype, indicating that plasmid-encoded RidA partially restores the growth phenotype of the *ridA* mutant. HOCl concentrations >2 mM led to a complete growth arrest, while concentrations <1 mM did not influence growth of either strain (Fig. 7). Our results indicate that RidA has a role in protecting *E. coli* cells against the consequences of HOCl-mediated stress and thus might be an integral component of the bacterial defense strategies against the mammalian immune system.

### Mass spectrometry identifies cytosolic clients of RidA

**Figure 8:**
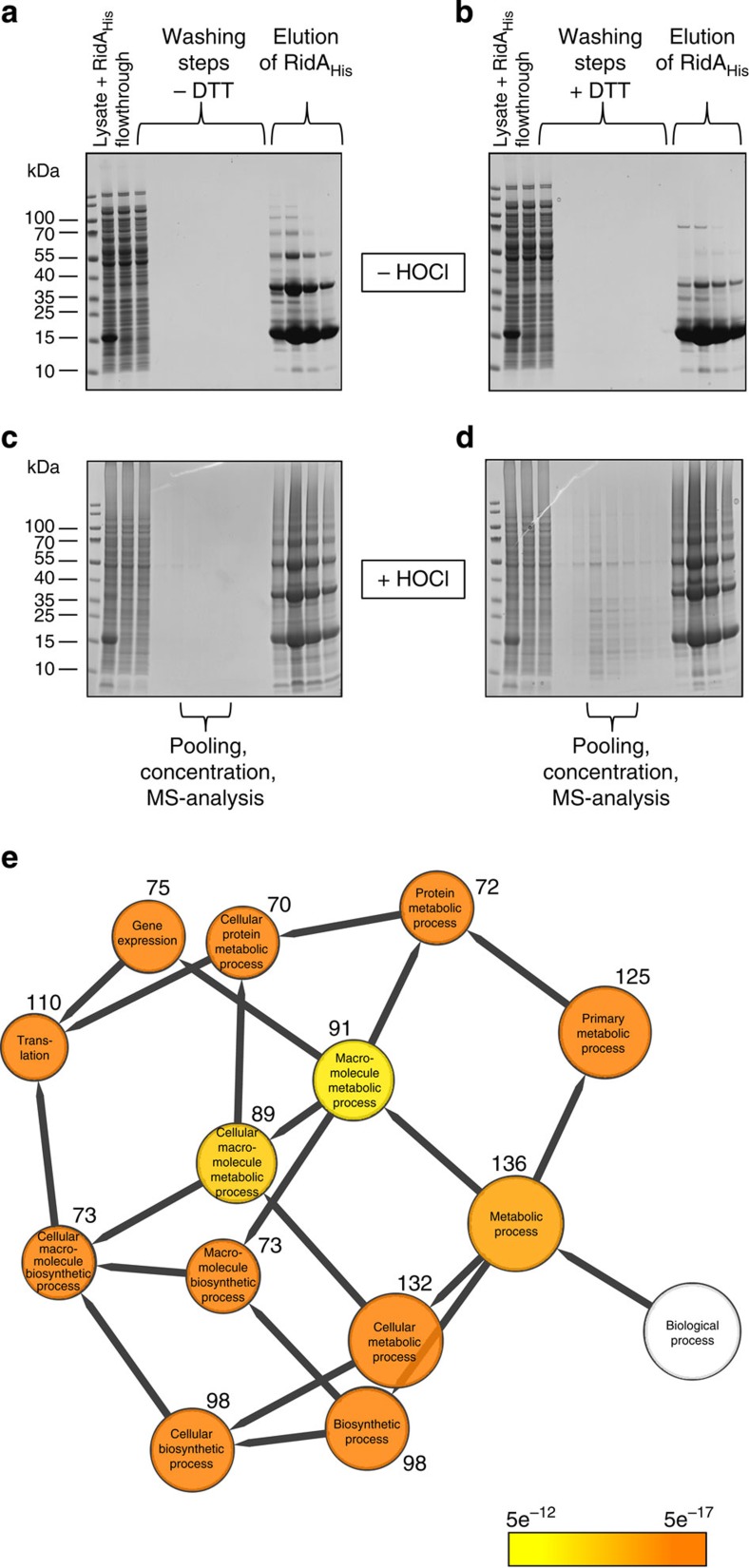
HOCl-treated RidA binds to chlorine-treated cytosolic *E. coli* proteins. (**a**) His-tagged RidA was spiked into *E. coli* MG1655 Δ*ridA* cell lysate. HOCl was added in **c** and **d** or left out in **a** and **b**. The mixtures were applied to four separate Ni^2+^-NTA chromatography columns. After intensive washing, DTT-containing buffer was used to elute proteins that were bound to RidA in **b** and **d**. Controls were washed with the same volumes of DTT-free washing buffer (shown in **a** and **c**). Elution of RidA_His_ was performed using buffer containing 250 mM of imidazole. Samples from each fraction were applied to non-reducing SDS gels. (**e**) Proteins were categorized into biological processes according to GO terms using the Cytoscape/BiNGO interface. The number of proteins found for each category is given. The *P* value of the enrichment of a certain category is indicated by colour.

To identify cytosolic RidA substrate proteins, we co-purified proteins from cell lysates bound to His-tagged RidA_HOCl_ by Ni^2+^-NTA chromatography and elution by de-chlorination (Fig. 8a–d). For this purpose, His-tagged RidA was added to *E. coli* MG1655-Δ*ridA* cell lysates. HOCl was added in a 10-fold excess over the final RidA_His_ concentration. Controls were left untreated. After loading the mixture onto Ni^2+^-NTA chromatography columns and extensive washing, DTT-containing buffer was used to reduce RidA and thereby elute bound substrate proteins (Fig. 8b,d). No significant elution was observed in the HOCl-free controls (Fig. 8a,b), as well as the DTT-free control (Fig. 8c).

Substrate proteins were identified by mass spectrometry after tryptic digest (Supplementary Tables 2,3). A total of 143 proteins were identified, 18 of which eluted exclusively in samples with DTT (Supplementary Table 3). The abundance of proteins was much higher in samples after DTT elution, except for the protein FklB. Sixty-five of the identified proteins belong to the most abundant proteins in *E. coli* MG1655 measured after growth in minimal medium^34^. Identified proteins from Supplementary Tables 2 and 3 were categorized according to their biological function using the program Cytoscape (Fig. 8e). This analysis confirmed that the majority of proteins binding to RidA_HOCl_ were high abundant proteins associated with the primary metabolism of *E. coli*. It is, therefore, most likely that RidA acts as an unspecific chaperone that binds any unfolding protein in its direct vicinity at the onset of chlorine stress. However, we also identified proteins that are not included in the dataset from Lu *et al*. and may therefore be of lower abundance, such as thioredoxin 1 (TrxA), uracil phosphoribosyltransferase Upp and iron sulfur cluster assembly scaffold protein IscU^34^. Whether such proteins are preferentially bound by *N*-chlorinated RidA remains subject to further studies.

## Discussion

RidA from *E. coli* belongs to the YjgF/YER057c/UK114 family of proteins with members in all domains of life. Although highly conserved, different functions have been assigned to members of this family in different species. In *Enterobacteriaceae*, RidA is an enamine/imine deaminase^21^. We recently reported that RidA is important to overcome nitrosative stress in *E. coli*^23^. Treatment of cells with peroxynitrite led to oxidation of the single cysteine residue of RidA. In this oxidized state, RidA was no longer able to accelerate the release of ammonia from an enamine/imine intermediate generated by threonine dehydratase IlvA. We then tested the influence of various other oxidative stressors on RidA’s enamine/imine deaminase function *in vitro*. To our surprise, HOCl treatment of RidA did not result in its inactivation but instead RidA bound tightly to IlvA, leading to the inhibition of IlvA’s dehydratase activity.

In the present study, we show that this inhibition is the result of a chaperone function of HOCl-activated RidA. Activation of RidA’s chaperone function by HOCl occurs rapidly within sample mixing time, similar to previous reports on Hsp33, another bleach-activated chaperone^16^. Activation was maximal at a 10-fold molar excess of HOCl over RidA. However, in contrast to the activation of other redox-regulated chaperones, such as Hsp33 or 2-cys peroxiredoxins, which involve cysteine oxidation^35^, activation of RidA is cysteine-independent. A cysteine-free RidA variant can still be fully activated by bleach. Activation of chaperone function coincides with a decrease in amino group content, an increase in hydrophobicity and the appearance of multiply chlorinated RidA species as shown by mass spectrometry (Fig. 5). We suspect that *N*-chlorination most likely occurs at the ε-amino groups of lysine and/or the guanidinium groups of arginine as well as the protein amino (N) terminus. The formation of chloramines has previously been regarded as a damaging amino acid modification, which could result in protein inactivation, unfolding, aggregation and even fragmentation due to halogen transfer and formation of cross-links^3^,^36^,^37^,^38^,^39^,^40^. However, we could show that chloramines of RidA are fully removed by treatment with DTT and ascorbic acid. This restoration is accompanied by the inactivation of RidA’s chaperone function. A new cycle of activation is possible after reduction, demonstrating that the reaction of RidA with HOCl and chloramines is fully reversible and does not alter the structural integrity of the protein.

*In vivo*, the lack of RidA makes *E. coli* more susceptible to chlorine stress. A knockout mutant in a BL21 strain background shows a significant growth deficiency in the presence of 2 mM HOCl. Expression of RidA from a plasmid could partially restore this phenotype. A full rescue is probably not achieved due to gene dosage effects and an overall increased stress level due to protein overexpression. In this specific assay, the BL21-Δ*hslO* strain (Hsp33 deficient) behaved wild-type-like and was less sensitive towards HOCl as compared with the BL21-Δ*ridA* mutant. This was surprising, as a Δ*hslO* strain has been shown before to be hypersensitive towards HOCl^16^. However, these experiments have been performed in the already chlorine-sensitive *E. coli* MC4100. Various attempts to construct a Δ*ridA* strain in the MC4100 strain, as well as in a Δ*hslO* background, (Δ*hslO*–Δ*ridA* double mutant) failed, indicating that the lack of *ridA* in these strains might be lethal.

Purification of RidA_HOCl_ substrate proteins from *E. coli* cell lysates after HOCl treatment revealed that highly abundant cytosolic proteins, such as ribosomal subunit proteins, are bound by RidA_HOCl_. A pathway analysis assigned the majority of these proteins to the primary metabolism. This finding, although performed in cell lysates, points towards an unspecific binding mechanism of *N*-chlorinated RidA *in vivo*. The identification of numerous cysteine-free proteins, such as the trigger factor, demonstrates that binding of *N*-chlorinated RidA does not depend on cysteine disulfide bond formation.

Looking for an *in vivo* mechanism of inactivation of RidA’s chaperone function, we found that catalytic quantities of a reconstituted *E. coli* thioredoxin system could inactivate RidA_HOCl_ NADPH-dependently *in vitro*. Rates observed in our assay were 10- to 20-fold lower than rates observed in a classic thioredoxin activity assay, which uses insulin as substrate but substantially higher than rates obtained with oxidized glutathione as substrate^41^. We assume that this inactivation occurs when the cell is no longer exposed to HOCl and once again enough reduction equivalents are available to the Trx system. The exact mechanism of the thioredoxin-dependent reduction has to be investigated in future experiments. It might be that chlorines from the amino groups in RidA are transferred to the thiol group of the attacking cysteine in TrxA’s active site. This would result in the formation of a sulfenyl chloride intermediate. The sulfenyl chloride would then be reduced by the resolving cysteine of TrxA’s active site and H^+^ and Cl^−^ will be released. The disulfide bond formed in this reaction may then become reduced NADPH-dependently by thioredoxin reductase TrxB. To our knowledge, the reduction of chloramines by thioredoxin has not been reported to date and is a new function of this ubiquitous reducing system. However, we also observed glutathione-based inactivation of RidA_HOCl_. As glutathione concentrations are in the millimolar range in the bacterial cytoplasm^42^, it may be assumed that inactivation by glutathione is a major mechanism of inactivation *in vivo*.

**Figure 9:**
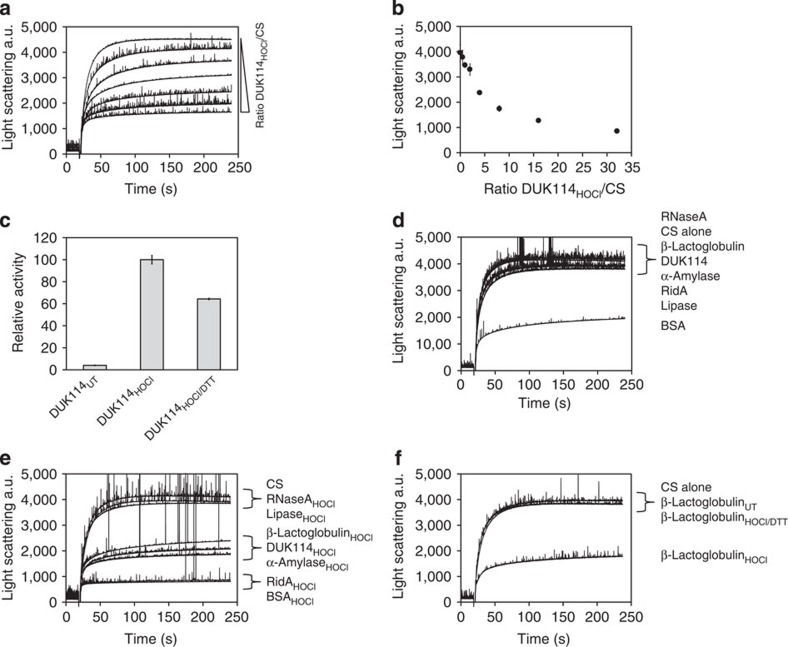
HOCl induces chaperone activity in homologues to RidA and unrelated proteins. (**a**,**b**) The *Drosophila melanogaster* RidA homologue DUK114 was purified and subjected to chaperone assays. Inhibition of citrate synthase aggregation strongly depends on the ratio of DUK114_HOCl_ over citrate synthase similar to RidA_HOCl_ and other unrelated proteins. (**c**) In contrast to RidA_HOCl_ (and β-lactoglobulin; see also **f**), activation of DUK114_HOCl_ is only partially reversible by DTT. (**d**,**e**) In addition to DUK114, a number of other proteins was tested for HOCl-induced chaperone activity. RNaseA and lipase are unaffected by HOCl treatment, whereas β-lactoglobulin, α-amylase and BSA show HOCl-inducible chaperone activity. BSA is already active before treatment (**d**). (**f**) Activation of β-lactoglobulin is reversible by DTT. Representative measurements are shown for **a**,**d**,**e** and **f**. Data represented in **b** and **c** are the means of three independent measurements.

We asked whether the observed chaperone activity is a shared characteristic of the whole YjgF/YER057c/UK114 family of proteins. We therefore analysed the *Drosophila melanogaster* RidA homologue DUK114. This protein has previously been shown to act as a chaperone, albeit no prior HOCl treatment was reported^43^. In our hands, untreated DUK114, heterologously expressed and purified from *E. coli*, could not inhibit citrate synthase aggregation (Fig. 9). However, treatment with HOCl induced holdase activity in DUK114, comparable to RidA. These seemingly contradictory findings might be explained by a different purification protocol used in our study, which included DTT during the preparation of the protein. DTT reduces the modifications that might have been introduced *in vivo*. However, DUK114_HOCl_ remained active after DTT treatment and still inhibited citrate synthase aggregation, indicating distinct mechanisms of activation and deactivation of YjgF/YER057c/UK114 family members.

We also found that a number of other proteins, including β-lactoglobulin, α-amylase and bovine serum albumin (BSA) showed HOCl-dependent holdase activity (Fig. 9). Chaperone activity of BSA has been reported before and we show that this activity is further increased by HOCl treatment^44^. Similarly, chaperone activity of human α_2_-macroglobulin has been shown to be largely increased by HOCl treatment^45^. Like BSA, α_2_-macroglobulin is a circulating blood plasma protein, indicating that a set of chaperones might be responsible for the protection of host proteins upon infection and inflammation.

Other proteins such as RNaseA and porcine pancreas lipase did not respond to HOCl treatment (Fig. 9), while lysozyme was highly prone to precipitation during treatment as has been reported before^46^. *N*-chlorination by HOCl has been investigated in more detail for various proteins including low-density lipoprotein, lysozyme and insulin^38^,^47^,^48^. The extent of *N*-chlorination differed significantly depending on parameters such as surface accessibility, presence of thiol-containing amino acids, the presence of histidine, and the overall tertiary structure of the protein^48^. Recently, the BSA homologue human serum albumin was shown to inhibit the major high-density lipoprotein receptor SR-BI^49^. Interestingly, this inhibition depends on *N*-chlorination of lysine residues and is irreversible. Moreover, HOCl-modified human serum albumin was shown to bind and inhibit proteins from West Nile virus and HIV^50^,^51^. These findings suggest a critical role of HOCl-modified plasma proteins during infection and inflammatory processes.

We compared the predicted physicochemical properties of these proteins (protein localization, number of specific amino acids, predicted hydrophobicity and isoelectric point) to identify possible characteristics that are favourable for turning a protein into a HOCl-mediated holdase (Supplementary Table 4). However, on the basis of these properties, a clear commonality between the HOCl-mediated holdase proteins could not be established.

In bacteria, together with other proteins, such as Hsp33, which is activated by disulfide bond formation and the novel HOCl-responsive transcription factors HypT, which is activated by methionine oxidation^12^,^13^, and NemR and RclR, which are activated by cysteine oxidation^14^,^15^, *N*-chlorinated RidA may protect cells during host invasion from the deleterious effect of the strong physiological oxidant hypochlorous acid.

**Figure 10:**
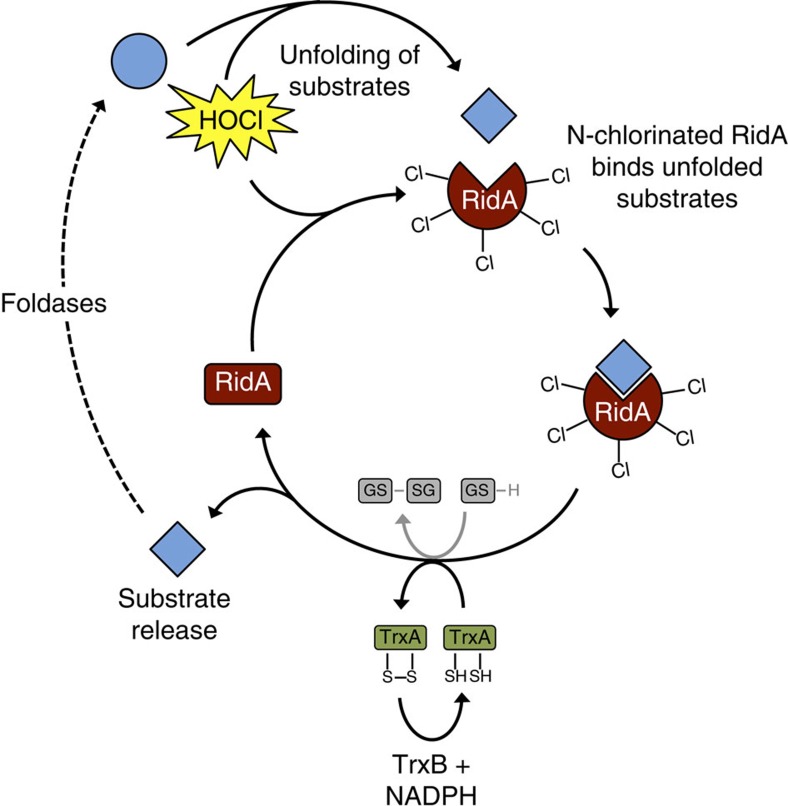
Chaperone activation/inactivation cycle of RidA. When bacterial cells are exposed to hypochlorous acid, cytoplasmic proteins unfold. At the same time, RidA is *N*-chlorinated. Under these conditions *N*-chlorinated RidA binds to a wide range of unfolded client proteins, preventing their aggregation. Upon decrease of hypochlorous acid in the cellular environment, the thioredoxin system and reduced glutathione (GSH) are able to remove chlorine from RidA, which leads to release of the client protein. The client protein can then be refolded.

*N*-chlorination of proteins constitutes a novel activity-modulating post-translational switch that reversibly turns RidA into a holdase. *N*-chlorinated RidA binds a wide spectrum of proteins and can prevent protein aggregation of model substrates, such as citrate synthase or IlvA. Removal of the chloramine modifications, which can be performed NADPH-dependently by catalytic amounts of the thioredoxin system, leads to substrate release by RidA. Foldases can then refold these proteins (Fig. 10).

## Methods

### Preparation of chlorinating agents

Concentration of NaOCl (Sigma-Aldrich, St. Louis, USA) was measured with a JASCO V-650 spectrophotometer (JASCO, Tokyo, Japan) at 292 nm using the extinction coefficient *ε*_292_=350 M^−1^ cm^−1^. Monochloramine and *N*-chlorotaurine were prepared freshly by mixing 200 mM NH_4_Cl or 200 mM taurine, respectively, solved in 0.1 M KOH with 200 mM NaOCl. Concentrations of products were measured at 242 and 251 nm using extinction coefficients *ε*_242_=429 M^−1^ cm^−1^ for monochloramine and *ε*_251_=397 M^−1^ cm^−1^ for *N*-chlorotaurine.

### Protein purification

Overexpression and purification of RidA, RidA mutant and IlvA was performed as previously described using strains and plasmids listed in Supplementary Table 1 (refs. 23, 52). Protein concentrations were determined using a JASCO V-650 spectrophotometer. Extinction coefficients used were *ε*_280_=2,980 M^−1^ cm^−1^ for the monomer of RidA, *ε*_280_=4,470 M^−1^ cm^−1^ for DUK114, and *ε*_280_=31,860 M^−1^ cm^−1^ for IlvA.

### Oxidation and reduction of proteins

Oxidation and reduction was performed by incubation of 700 μM of the respective protein with varying ratios of HOCl, monochloramine, DTT or ascorbic acid for incubation times indicated at 30 and 37 °C, respectively. Removal of stressors was carried out with Micro Bio-Spin Chromatography Columns according to the manufacturer’s instructions (Bio-Rad, München, Germany).

### Aggregation assays with citrate synthase and IlvA

To monitor aggregation, 12 μM citrate synthase or IlvA were chemically denatured in 4.5 M GdnCl, 40 mM HEPES, pH 7.5 at room temperature overnight. Aggregation was induced by the addition of 0.15 μM citrate synthase or 0.3 μM IlvA to 1,600 μl 40 mM HEPES, pH 7.5. Increase of light scattering was monitored with a JASCO FP-8500 fluorescence spectrometer equipped with a Peltier thermo-holder ‘EHC-813’ at 30 °C for 240 s under continuous stirring. Measurement parameters were set to 360 nm (Ex/Em), 2.5 nm slit width (Ex/Em) and medium sensitivity. The activity of chaperones was determined by the addition of varying amounts of untreated or treated proteins before the addition of citrate synthase or IlvA. Calculation of relative chaperone activities was performed by the determination of initial and final absorbance of individual samples and correlation to maximal inhibition of light scattering. For thermal aggregation assays, 10 μM of untreated or HOCl-treated RidA were added to 1,600 μl of prewarmed 40 mM HEPES, pH 7.5. Measurement parameters were set to 360 nm (Ex/Em), 2.5 nm slit width (Ex/Em) and medium sensitivity. Measurements were performed at 43 °C for 1,200 s under continuous stirring. After 60 s, citrate synthase was added to a final concentration of 0.3 μM. To monitor citrate synthase aggregation upon release by RidA_HOCl_, 5 mM DTT was added to the cuvette after 250 s of measurement.

To measure the influence of ATP on chaperone activity, a twofold or eightfold excess of RidA over citrate synthase was used. ATP was added in a 10-fold excess over RidA.

### Thioredoxin- and glutathione-based inactivation of RidA_HOCl_

Reduction of RidA by thioredoxin was followed spectrometrically at the absorption maximum of NADPH using a JASCO V-650 spectrophotometer equipped with the temperature-controlled cell holder PSC-718. An amount of 50 μM RidA were mixed with 5 μM TrxA (Sigma-Aldrich, St Louis, USA) and 100 μM NADPH (Sigma-Aldrich, St. Louis, USA) in 50 mM Tris-buffer, pH 7.5. After 30 s of stirring, 80 nM TrxB were added. Consumption of NADPH was measured at 340 nm for 2 h.

Reduction by glutathione was carried out by incubating RidA_HOCl_ with a 1-fold or 10-fold molar excess of glutathione (GSH or GSSG) for 30 min at 37 °C before the measurement of chaperone holdase activity.

### Detection of accessible amino groups—fluorescamine assay

The detection of protein amino groups was carried out as previously described^32^. Briefly, 3 mg ml^−1^ of fluorescamine (Sigma-Aldrich, St Louis, USA) were dissolved in acetone. One millilitre of 100 μg ml^−1^ RidA solutions in phosphate-buffered saline were mixed with 334 μl of fluorescamine solution. Emission of fluorescamine was measured from 400 to 600 nm with the excitation set to 388 nm. Relative amino group content was calculated by setting the fluorescence of fully reduced RidA to 100%.

### Nile red hydrophobicity assays of RidA and RidA_HOCl_

Nile red (Sigma-Aldrich, St. Louis, USA) was dissolved in DMSO to 0.25 mM. Varying concentrations of RidA and RidA_HOCl_ (1–200 μM) were mixed in 40 mM HEPES-KOH, pH 7.4 with 0.2 μM Nile red. The fluorescence was monitored using a JASCO FP-8500 fluorescence spectrometer equipped with a Peltier thermo-holder ‘EHC-813’. Measurement parameters were set to 550 nm excitation, 570–700 nm emission with 5 nm slit width (Ex/Em) and medium sensitivity. RidA and RidA_HOCl_ concentrations resulting in half-saturation were calculated from these data using Igor Pro Version 6.34A (Wave Metrics, Lake Oswego, USA) and Excel for Mac 2011 version 14.3.9 (Microsoft, Redmond, USA). Data were fitted with Igor Pro using the ‘Sigmoid’ fit function. To obtain the negative inverse of the half-saturation, the reverse protein concentration was plotted against the reverse fluorescence intensity and the ‘Trendline’ linear regression function of Excel was used.

### Mass spectrometry of full-length RidA

RidA treated with HOCl and MCA, and/or the reductant DTT was adjusted for pipette tip-based purification to 0.1% TFA. Purification was performed using OMIX C18 Tips (Varian, Walnut Creek, USA) according to the manufacturer’s instructions. Elution of RidA was performed with 100 μl 0.1% FA, 70% acetonitrile. RidA was then directly injected into an Orbitrap Elite mass spectrometer (Thermo Scientific, Waltham, USA) using an API Source with a static nanospray probe and a 1 μm picoTip emitter from EconoTips (NewObjective, Woburn, USA). Measurements were performed with a source voltage of 1.2 kV and a capillary temperature of 275 °C. Spectra were deconvoluted and further analysed using Xcalibur software (Thermo Scientific, Waltham, USA).

### Threonine deaminase assay

Analysis of α-ketobutyrate generation was performed as described previously^21^,^23^. Briefly, RidA was treated with HOCl and/or DTT as described in the main body of the manuscript. After the removal of reagents, 1.8 μM RidA were mixed with 0.9 μM IlvA in 1 × reaction buffer (50 mM MES, 50 mM HEPES, 50 mM TAPS pH 7.5). The reaction was started by the addition of 10 mM threonine. α-Ketobutyrate generation was followed at 230 nm in a JASCO V-650 spectrophotometer equipped with the temperature-controlled cell holder PSC-718 (JASCO, Tokyo, Japan) using a 3.5 ml QS-macro-cuvette (10 mm) with a magnetic stir bar at 20 °C.

### Oxidation of RidA by different oxidants

Treatment of RidA with peroxynitrite, H_2_O_2_, H_2_O_2_/Cu was performed using a 10-fold excess of the oxidants. After 10 min of incubation at 30 °C, oxidants were removed using Micro Bio-Spin Chromatography Columns according to the manufacturer’s instructions (Bio-Rad, München, Germany).

### Analytical gel filtration

Analytical gel filtration was carried out as described previously^23^. Briefly, untreated or HOCl-treated RidA was loaded to a Superdex 75 10/300 GL column connected to an ÄKTA purifier FPLC system, which was set to a flow rate of 0.5 ml min^−1^.

### Chaperone assays with DUK114 and unrelated proteins

Bovine serum albumin (from bovine serum), lipase (from porcine pancreas), ribonuclease A (from bovine pancreas), lysozyme (from chicken egg white), α-amylase (from porcine pancreas) and β-lactoglobulin were purchased from Fluka (Seelze, Germany) or Sigma-Aldrich (St. Louis, USA). DUK114 (*Drosophila melanogaster*) was purified using the same protocol as for RidA^23^. Proteins were dissolved in 0.1 M KH_2_PO_4_, pH 8.0, 1 mM EDTA and treated with a 10-fold excess of HOCl for 10 min at 30 °C. HOCl was removed using Micro Bio-Spin Chromatography Columns according to the manufacturer’s instructions (Bio-Rad, München, Germany).

### Construction of *E. coli* deletion strains

*E. coli* BL21-Δ*hslO* was constructed by P1 transduction of the corresponding gene knockouts from the Keio Collection from the National BioResource Project (National Institute of Genomics, Japan)^53^. P1 transduction was performed as described earlier^54^. The successful transduction was verified by PCR using strain-specific primers and kanamycin-resistant cassette-specific primers^55^ (Supplementary Table 1).

### Cultivation of *E. coli* and stress treatment

*E. coli* strains BL21, CL048 (BL21-Δ*ridA*) and CL053 (BL21-pET11-a_*ridA*) were grown in LB medium at 37 °C to OD_600_ of 0.3. Overexpression of RidA in CL053 was induced by the addition of 1 mM IPTG for 30 min. Cells were OD_600_-normalized and inoculated to OD_600_=0.1 into microtiter plates filled with serial 1:1 dilutions of HOCl-containing LB starting with 32 mM NaOCl. Growth of strains at 37 °C was monitored in an Infinite 200 PRO Microplate Reader (TECAN, Männedorf, Switzerland).

### Purification of RidA substrate proteins

RidA substrate proteins were purified by Ni-NTA chromatography. For this purpose, His-tagged RidA (RidA_His_) was overexpressed in *E. coli* BL21_Δ*ridA* (strain AM01; Supplementary Table 1) and purified by Ni^2+^-NTA chromatography as previously described for IlvA^21^. Purified RidA_His_ was added to *E. coli* MG1655_Δ*ridA* cell lysates to a final concentration of 1.4 mM. This mixture was either left untreated or was treated with 14 mM HOCl. Cell lysates were loaded onto pre-equilibrated Ni^2+^-NTA chromatography columns (Qiagen; 1 ml bed volume). After collecting the flow-through, columns were washed with 20 ml washing buffer (50 mM NaH_2_PO_4_, 300 mM NaCl, 20 mM imidazole, pH 8.0). Elution of substrate proteins was achieved by four additional washing steps with washing buffer containing 10 mM DTT. After washing two times with DTT-free buffer, RidA_His_ was eluted with elution buffer (50 mM NaH_2_PO_4_, 300 mM NaCl, 250 mM imidazole, pH 8.0). Samples were analysed on non-reducing SDS gels. After pooling and concentrating of washing fractions four to six, protein identification was performed by mass spectrometry as described below.

### Tryptic digestion and nanoUPLC

A volume of 25 μl of each sample containing 0.1% RapiGest (Waters, Eschborn, Germany) was subjected to tryptic digestion. Cysteine reduction using 2.5 mM TCEP (Invitrogen, Carlsbad, USA) for 60 min at 60 °C was followed by alkylation with 5 mM iodoacetamide (Sigma-Aldrich, St Louis, USA) for 15 min at 25 °C in the dark and addition of trypsin to a final concentration of 5 ng μl^−1^ (Sequencing grade; Promega, Madison, USA). After incubation with mild shaking at 37 °C for 5 h, 2 μl of concentrated TFA (AppliChem, Darmstadt, Germany) was added to the sample and precipitated RapiGest was removed by centrifugation. PhosB peptides as Hi3 quantification standard (Waters) were added to a final concentration of 12.5 fmol μl^−1^. Samples were injected into a nanoAcquity UPLC system to be loaded on a trap column (C18, pore size 100 Å, particle diameter 5 μm, inner diameter 180 μm, length 20 mm) and were then eluted using a gradient (350 μl min^−1^, linear gradient 0.5–5% in 2 min, 5–70% in 82 min) of acetonitrile with 0.1% formic acid (Biosolve, Valkenswaard, Netherlands) from an analytical column at 40 °C (C18, pore size 130 Å, particle diameter 1.7 μm, inner diameter 75 μm, length 150 mm).

### Mass spectrometry

The nanoUPLC column was coupled online to a Synapt G2-S HDMS ESI/ToF mass spectrometer (Waters). Spectra were recorded in positive ionization mode and resolution mode over a mass range of 50–1,800 *m*/*z* with 0.5 s per scan using the MS^E^ technology and a trap collision energy ramp of 14–45 V. The following parameters were used for the NanoLockSpray source: capillary voltage, 2.0 kV; sampling cone voltage, 30 V; source offset, 30 V; source temperature, 100 °C; desolvation temperature, 150 °C; cone gas flow; 50 l h^−1^; nanoflow gas pressure, 0.5 bar; desolvation gas flow, 500 l h^−1^. Leucine enkephalin serving as lock mass analyte was fed through the lock spray channel (lock mass capillary voltage, 3.0 kV). Analysis of the spectra was performed using MassLynx V4.1 SCN813 (Waters).

### Data analysis

Data were analysed using the ProteinLynx Global Server 2.5.2 (Waters) software. Mass spectra were processed using the following parameters: chromatographic peak width, automatic; MS ToF resolution, automatic; lock mass window, 0.25 Da; low energy threshold, 50 counts; elevated energy threshold, 15 counts; intensity threshold, 500 counts. A non-redundant version of the *Escherichia coli* MG1655 database (NCBI accession NC_000913.3) containing 4,109 protein entries (including sequences of the PhosB standard, trypsin, keratin and RidA-His_6_) was used for protein identification using the following parameters: peptide tolerance, automatic; fragment tolerance, automatic; minimal fragment ion matches per peptide, 2; minimal fragment ion matches per protein, 2; minimal peptide matches per protein, 1; maximum protein mass, 300 kDa; primary digest reagent, trypsin; secondary digest reagent, none; missed cleavages, 2; fixed modifications, carbamidomethyl C; variable modifications, deamidation N and Q, oxidation M, oxidation C, sulfinic acid C, cysteic acid C; false positive rate, 4%; calibration protein, PhosB standard. Only proteins identified with at least three peptides in at least two of three replicates were considered for evaluation.

### Gene ontology of RidA interaction partners

Proteins that were eluted after DTT treatment were categorized according to biological functions. The EcoCyc GO annotation of *E.coli*^56^ served as database using the program Cytoscape (version 2.8.2, ref. 57) with the BiNGO plugin (version 2.44, ref. 58) and allowed categorization of 140 out of 144 proteins (except for FkbA, YifE, HupB, RhmD) using the following settings: statistical test, hypergeometric test; multiple testing correction, Benjamini & Hochberg FDR correction; reference set, whole annotation.

## Author contributions

A.M. with L.I.O.L. designed and conceived the experiments and wrote the manuscript. A.M. performed the chaperone assays, thioredoxin and glutathione reduction assays, measurement of amino group contents, hydrophobicity assays, purification of RidA substrates, growth experiments and gel filtration. A.M. and L.I.O.L. performed mass spectrometry of full-length RidA. N.L. performed threonine deaminase assays and purified RidA and DUK114. A.M. and S.L. identified RidA substrate proteins by mass spectrometry. J.E.B., S.L. and K.K. evaluated the MS data and set up MS-based experimental procedures. S.L. performed the GO annotation of RidA clients.

## Additional information

**How to cite this article:** Müller, A. *et al*. Activation of RidA chaperone function by *N*-chlorination. *Nat. Commun.* 5:5804 doi: 10.1038/ncomms6804 (2014).

## Supplementary Material

Supplementary Information Supplementary Tables 1-4 and Supplementary References
