# Supplementary material for: Activation of RidA chaperone function by N-chlorination
Source: Nat Commun. 2014 Dec 17;5:5804. doi: 10.1038/ncomms6804 (PMC4284807; doi:10.1038/ncomms6804)
Supplement: Supplementary Information — Supplementary Tables 1-4 and Supplementary References [file ncomms6804-s1.pdf]

SUPPLEMENTARY TABLES

Supplementary Table 1: Primers and *Escherichia coli* strains used in this study

**Primer**	**Sequence (5' - 3')**	**Source**
hslo-fw	CGACCTGTTACGATTTAAACACGGC	This study
hslO-rv	CGTAAAATCCGGCAGAGCCC	This study
K1	CAGTCATAGCCGAATAGCCT	[1]
K2	GGTGCCCTGAATGAACTGC	[1]
**Strain**	**Genotype**	**Source**
BL21 (DE3)	F^- *ompT gal dcm lon hsdS*$_B$(r_B^- m_B^-) λ(DE3 [*lacI lacUV*5-T7 gene 1 *ind1 sam7 nin5*])	Stratagene, Santa Clara, CA
CL048	BL21 (DE3) *ridA*::Kan	[2]
CL053	BL21 (DE3) pCL001 (RidA in pET11-a)	[2]
CL054	CL048 pCL002 (RidA Cys107Ser in pET11-a)	[2]
AM01	BL21 (DE3) *ridA*::Kan (RidA in pET22-b)	This study
NL59	BL21 (DE3) (IlvA in pET22-b)	This study
NL60	CL048 (DUK114 in pET11-a)	This study
AM02	BL21 (DE3) *hslO*::Kan	This study

Supplementary Table 2: RidA substrate proteins identified from HOCl-treated and untreated cell lysates

GenBank ID	Protein function	Ratio +DTT/Control[a]	Mean protein quantity (fmol)[b]		Standard deviation[b]		Abundance ranking[c]
			**Control**	**+DTT**	**Control**	**+DTT**	
1790433	HupA HU DNA binding transcriptional regulator alpha subunit	574.79	2.13	1225.00	0.56	1065.00	45
1789698	RpmD 50S ribosomal subunit protein L30	511.49	4.58	2343.09	2.06	1736.08	87
1786206	RpsT 30S ribosomal subunit protein S20	196.80	4.40	866.13	3.72	539.22	75
1789702	RpsH 30S ribosomal subunit protein S8	132.44	7.04	932.53	2.35	647.42	109
1786822	AhpC alkyl hydroperoxide reductase C22 subunit	126.29	0.65	82.21	0.01	59.84	13
1786644	HupB HU DNA binding transcriptional regulator beta subunit	123.35	0.78	95.96	0.51	79.37	112
1790067	RpmG 50S ribosomal subunit protein L33	117.06	2.77	324.63	2.93	173.89	53
2367334	RplK 50S ribosomal subunit protein L11	109.83	33.50	3679.50	13.47	2231.20	61
1790647	RplI 50S ribosomal subunit protein L9	107.36	9.78	1050.22	5.24	853.03	30
1788126	CspC stress protein member of the CspA family	89.02	11.05	983.72	5.71	812.89	7
1790417	RplJ 50S ribosomal subunit protein L10	88.84	17.15	1523.30	6.10	1157.60	111
1790416	RplA 50S ribosomal subunit protein L1	88.11	22.91	2018.28	10.81	1216.66	49
1786841	CspE DNA binding transcriptional repressor	79.66	8.21	654.07	3.75	530.85	56
1790585	GroS Cpn10 chaperonin GroES small subunit of GroESL	78.13	3.14	245.46	1.06	212.54	4
1789699	RpsE 30S ribosomal subunit protein S5	74.72	18.04	1348.24	4.87	347.70	11
1788012	InfC protein chain initiation factor IF 3	68.00	7.08	481.30	0.76	254.73	118
1786351	ErpA iron sulfur cluster insertion protein	65.86	2.49	164.06	0.83	21.24	244
1786351	RplW 50S ribosomal subunit protein L23	65.62	6.10	400.39	0.79	235.65	8
1787335	FabG 3 oxoacyl acyl carrier protein reductase	61.91	1.35	83.76	0.53	62.88	201
1788855	GuaB IMP dehydrogenase	60.99	0.61	37.44	0.27	29.68	123
1789700	RplR 50S ribosomal subunit protein L18	58.85	10.03	590.55	2.56	407.91	100
1788958	RplS 50S ribosomal subunit protein L19	58.67	22.46	1318.09	8.92	668.89	33
1789577	RplU 50S ribosomal subunit protein L21	56.67	16.14	914.73	8.43	947.93	79
1789705	RplX 50S ribosomal subunit protein L24	56.16	9.07	509.41	6.58	424.83	25
1789697	RplO 50S ribosomal subunit protein L15	51.45	10.72	551.50	3.24	378.48	101
1788160	PykA pyruvate kinase II	51.34	2.04	104.99	0.54	80.32	370
1789446	RpsU 30S ribosomal subunit protein S21	48.51	5.57	269.99	1.20	159.61	79
1789716	RplC 50S ribosomal subunit protein L3	48.20	37.33	1799.33	16.96	1387.12	39
1789559	InfB fused protein chain initiation factor 2 IF2	48.05	1.31	62.81	0.17	5.80	217

GenBank ID	Protein function	Ratio +DTT/Control[a]	Mean protein quantity (fmol)[b]		Standard deviation[b]		Abundance ranking[c]
			Control	+DTT	Control	+DTT	
1789711	RplV 50S ribosomal subunit protein L22	46.20	4.19	193.73	2.75	54.62	18
1787336	AcpP acyl carrier protein ACP	45.69	5.23	239.14	2.19	36.33	3
1786196	DksA DNA binding transcriptional regulator of rRNA transcription DnaK suppressor	44.16	5.97	263.61	3.67	203.31	117
1789701	RplF 50S ribosomal subunit protein L6	43.55	35.90	1563.79	8.77	1011.56	21
1788755	PtsH phosphohistidinoprotein hexose phosphotransferase component of PTS system Hpr	42.17	3.23	136.18	0.57	110.71	NA[d]
1788079	GapA glyceraldehyde 3 phosphate dehydrogenase A	42.10	3.49	146.92	1.07	111.09	5
1788512	RplY 50S ribosomal subunit protein L25	41.33	3.03	125.02	1.06	101.74	22
1789141	Eno enolase	39.89	3.94	157.20	1.38	122.21	66
1789712	RpsS 30S ribosomal subunit protein S19	38.67	3.42	132.36	2.10	41.97	62
1788967	GrpE heat shock protein	38.11	1.27	48.59	0.16	59.07	160
1786640	Tig peptidyl prolyl cis trans isomerase trigger factor	37.71	6.35	239.28	2.30	181.48	73
1788757	Crr glucose specific enzyme IIA component of PTS	36.15	1.58	57.12	0.18	5.50	77
1786366	Tsf protein chain elongation factor EF Ts	36.13	4.64	167.61	1.75	99.72	44
1789703	RpsN 30S ribosomal subunit protein S14	35.80	1.92	68.65	0.98	20.39	159
1788005	IhfA integration host factor IHF DNA binding protein alpha subunit	35.06	1.52	53.22	0.57	25.66	190
1788473	MglB methyl galactoside transporter subunit	32.45	1.40	45.39	0.60	33.28	323
1789704	RplE 50S ribosomal subunit protein L5	32.24	11.43	368.47	4.28	261.34	36
1790068	RpmB 50S ribosomal subunit protein L28	32.15	5.54	178.23	1.58	30.98	80
1789717	RpsJ 30S ribosomal subunit protein S10	32.03	68.00	2178.20	12.10	1301.88	35
1789692	RpsK 30S ribosomal subunit protein S11	31.10	5.98	185.84	3.19	57.73	16
1790590	Efp polyproline specific translation elongation factor EF P	29.99	2.04	61.32	0.77	47.20	204
1790586	GroL Cpn60 chaperonin GroEL large subunit of GroESL	28.53	8.67	247.26	1.25	171.84	64
1787965	PykF pyruvate kinase I	28.06	4.01	112.48	1.77	87.25	161
1786362	DapD 2 3 4 5 tetrahydropyridine 2 carboxylate N succinyltransferase	27.80	0.84	23.27	0.61	4.05	65
1790835	PrfC peptide chain release factor RF 3	27.77	2.62	72.87	1.67	7.05	NA[d]
1786948	SucC succinyl CoA synthetase beta subunit	27.22	2.27	61.67	0.81	45.20	86
1786368	Frr ribosome recycling factor	26.06	3.45	90.02	2.38	16.86	144
1789576	RpmA 50S ribosomal subunit protein L27	25.65	5.69	145.97	1.63	89.21	NA[d]
87082118	Upp uracil phosphoribosyltransferase	25.55	3.31	84.48	0.46	51.85	NA[d]

GenBank ID	Protein function	Ratio +DTT/Control[a]	Mean protein quantity (fmol)[b]		Standard deviation[b]		Abundance ranking[c]
			Control	+DTT	Control	+DTT	
1787423	MinD membrane ATPase of the MinC MinD MinE system	25.23	0.57	14.38	0.02	14.88	371
1789740	RpsL 30S ribosomal subunit protein S12	24.76	2.05	50.69	0.65	19.13	NA[d]
1787489	Hns global DNA binding transcriptional dual regulator H NS	24.44	1.28	31.35	0.47	16.41	37
1789294	Pgk phosphoglycerate kinase protein	24.43	2.32	56.78	0.28	43.06	47
1789707	RpsQ 30S ribosomal subunit protein S17	22.85	3.92	89.64	0.56	52.91	NA[d]
1788663	FabB 3 oxoacyl acyl carrier protein synthase I	22.30	3.70	82.44	1.06	62.26	42
1788949	RaiA cold shock protein associated with 30S ribosomal subunit	22.26	3.86	85.97	1.10	63.44	NA[d]
2367114	CspG cold shock protein homolog cold inducible	22.21	0.92	20.50	1.06	30.49	NA[d]
1788635	Pta phosphate acetyltransferase	21.76	2.09	45.54	0.67	33.34	340
1789739	RpsG 30S ribosomal subunit protein S7	21.29	4.23	90.02	1.71	59.26	26
1789715	RplD 50S ribosomal subunit protein L4	21.25	21.44	455.61	5.10	246.01	48
1790844	DeoD purine nucleoside phosphorylase	21.14	3.71	78.48	1.45	60.09	NA[d]
1786365	RpsB 30S ribosomal subunit protein S2	20.86	68.90	1437.21	29.14	906.72	55
1788878	IscU iron sulfur cluster assembly scaffold protein	20.47	2.23	45.65	0.82	14.91	NA[d]
87081689	Hpt hypoxanthine phosphoribosyltransferase	20.00	2.38	47.61	0.37	8.55	NA[d]
29604058 6	RpsF 30S ribosomal subunit protein S6	19.52	10.00	195.13	8.12	152.05	50
1787488	GalU glucose 1 phosphate uridylyltransferase	19.43	0.97	18.87	0.19	1.73	261
1789819	NfuA Fe S biogenesis protein possible scaffold chaperone for damaged Fe S proteins	19.40	1.46	28.28	0.18	3.69	127
22651096 5	RpsI 30S ribosomal subunit protein S9	18.06	3.94	71.10	2.28	4.59	85
1789738	FusA protein chain elongation factor EF G GTP binding	17.78	5.73	101.82	2.82	75.74	14
1789691	RpsD 30S ribosomal subunit protein S4	17.08	9.40	160.53	3.65	86.68	31
1790217	Rho transcription termination factor	16.76	4.64	77.86	0.52	46.95	134
1789706	RplN 50S ribosomal subunit protein L14	16.54	9.98	164.98	1.69	54.04	116
1789693	RpsM 30S ribosomal subunit protein S13	15.71	4.80	75.37	0.97	49.81	12
1788756	PtsI PEP protein phosphotransferase of PTS system enzyme I	15.38	1.81	27.91	0.53	15.19	143
1790418	RplL 50S ribosomal subunit protein L7 L12	14.92	29.27	436.56	3.31	250.31	1
1789265	YgfZ iron sulfur cluster repair protein plumbagin resistance	14.03	3.13	43.95	0.71	31.43	213
1789710	RpsC 30S ribosomal subunit protein S3	12.83	11.40	146.19	1.54	21.69	38

GenBank ID	Protein function	Ratio +DTT/Control[a]	Mean protein quantity (fmol)[b]		Standard deviation[b]		Abundance ranking[c]
			Control	+DTT	Control	+DTT	
1789709	RplP 50S ribosomal subunit protein L16	12.79	6.72	85.97	3.38	58.53	82
1787140	RpsA 30S ribosomal subunit protein S1	12.76	5.40	68.83	0.72	43.01	24
1787131	PflB pyruvate formate lyase I	12.57	3.89	48.85	1.79	24.87	125
1789626	RplM 50S ribosomal subunit protein L13	11.62	14.45	167.99	7.77	40.33	19
16130606	RecA DNA strand exchange and recombination protein with protease and nuclease activity	11.37	2.71	30.79	1.16	7.01	299
87082390	FklB FKBP type peptidyl prolyl cis trans isomerase rotamase	11.23	1.52	17.04	0.22	10.17	NA[d]
1786307	Lpd lipoamide dehydrogenase E3 component is part of three enzyme complexes	11.14	12.12	134.98	1.88	61.43	95
48994988	TypA GTP binding protein	11.11	5.15	57.21	1.52	28.88	NA[d]
1788961	RpsP 30S ribosomal subunit protein S16	10.80	20.79	224.47	19.55	97.85	9
48994898	IscS cysteine desulfurase tRNA sulfurtransferase PLP dependent	10.79	3.30	35.61	1.29	9.62	NA[d]
2367097	AcnB bifunctional aconitate hydratase 2 2 methylisocitrate dehydratase	10.76	3.58	38.55	1.04	9.62	74
1789566	GlmM phosphoglucosamine mutase	10.68	3.04	32.48	1.40	16.49	304
1789142	PyrG CTP synthetase	10.56	2.66	28.09	0.69	12.92	NA[d]
1789690	RpoA RNA polymerase alpha subunit	10.54	5.18	54.56	2.05	38.11	32
1788633	AckA acetate kinase A and propionate kinase 2	9.98	9.44	94.21	4.21	61.50	229
1789311	MetK S-adenosylmethionine synthetase	9.52	4.13	39.29	1.36	17.26	63
1789689	RplQ 50S ribosomal subunit protein L17	9.50	17.20	163.39	4.03	123.74	59
1786305	AceF pyruvate dehydrogenase dihydrolipoyltransacetylase component E2	9.30	7.64	71.05	2.64	12.56	133
1790646	RpsR 30S ribosomal subunit protein S18	9.24	2.75	25.37	0.32	15.50	78
1790170	AtpD F1 sector of membrane bound ATP synthase beta subunit	9.12	4.53	41.26	1.00	27.19	135
1786196	DnaK chaperone Hsp70 co chaperone with DnaJ	8.94	7.65	68.41	4.48	39.89	51
1789560	NusA transcription termination antitermination L factor	8.79	1.10	9.70	0.17	6.91	252
1790038	SecB protein export chaperone	8.54	6.21	53.04	1.75	37.16	54
1790494	Ssb single stranded DNA binding protein	8.07	2.48	20.00	1.40	22.42	NA[d]
87082323	TnaA tryptophanase L cysteine desulfhydrase PLP dependent	7.51	16.41	123.28	7.89	42.37	NA[d]
1789737	TufA protein chain elongation factor EF Tu duplicate of tufB	7.48	283.18	2116.84	165.04	3176.35	NA[d]
1789654	AccC acetyl CoA carboxylase biotin carboxylase subunit	7.44	6.01	44.75	0.29	21.22	236
2367226	GlpD sn glycerol 3 phosphate dehydrogenase aerobic FAD NADP binding	7.22	7.88	56.84	2.83	40.11	NA[d]
1788825	Bcp peroxiredoxin thiol peroxidase thioredoxin dependent	6.32	3.39	21.41	0.93	18.98	88

GenBank ID	Protein function	Ratio +DTT/Control[a]	Mean protein quantity (fmol)[b]		Standard deviation[b]		Abundance ranking[c]
			**Control**	**+DTT**	**Control**	**+DTT**	
1787333	FabH 3 oxoacyl acyl carrier protein synthase III	5.97	3.24	19.36	0.73	11.32	198
90111711	RidA conserved protein UPF0131 family	5.34	1293.32	6900.36	578.58	1622.31	NA[d]
87082331	TrxA thioredoxin 1	4.26	2.32	9.89	2.83	6.99	NA[d]
1786304	AceE pyruvate dehydrogenase decarboxylase component E1 thiamin binding	3.61	24.87	89.82	12.50	60.13	110
1789713	RplB 50S ribosomal subunit protein L2	3.59	6.00	21.56	2.90	11.01	43
1790361	GlpK glycerol kinase	2.84	5.33	15.13	1.45	6.13	NA[d]
1790167	GlmS L glutamine D fructose 6 phosphate aminotransferase	2.44	12.41	30.31	4.47	3.08	256
1789556	RpsO 30S ribosomal subunit protein S15	2.32	108.22	251.32	74.43	124.57	60
1786899	Fur DNA binding transcriptional dual regulator of siderophore biosynthesis	1.49	43.77	65.10	2.82	6.95	238
606282	SlyD FKBP type peptidyl prolyl cis trans isomerase rotamase	0.84	22.04	18.62	2.64	7.83	103

[a] Ratios were calculated from mean protein quantities

[b] Mean protein quantities and standard deviations were calculated from at least two independent biological replicates

[c] Abundance ranking was calculated from[3]

[d] Abundance was not determined in[3]

Supplementary Table 3: RidA substrate proteins exclusively identified from HOCl-treated cell lysates

GenBank ID	Protein function	Mean protein quantity (fmol) in +DTT samples[a]	Standard deviation[a]	Abundance ranking[b]
226510960	RhmD L rhamnonate dehydratase	156.99	53.86	NA[c]
2367109	RpmE 50S ribosomal subunit protein L31	83.50	13.72	220
87081737	YajQ phage Phi6 host factor ATP GTP binding protein	77.87	59.03	NA[c]
1788655	AccD acetyl CoA carboxylase beta carboxyltransferase subunit	64.41	9.98	275
1787952	RibE riboflavin synthase beta chain	40.02	34.53	NA[c]
1789745	FkpA FKBP type peptidyl prolyl cis trans isomerase rotamase	39.46	7.54	174
1790366	HslU molecular chaperone and ATPase component of HslUV protease	37.89	26.44	295
1788546	RcsB DNA binding response regulator in two component regulatory system with RcsC	25.95	20.46	230
1786238	SurA peptidyl prolyl cis trans isomerase PPIase	25.78	5.12	333
1788877	IscA FeS cluster assembly protein	21.89	8.91	NA[c]
1790211	PpiC peptidyl prolyl cis trans isomerase C rotamase C	18.61	4.23	NA[c]
1786356	DegP serine endoprotease protease Do membrane associated	18.24	13.79	233
1788409	GatB galactitol specific enzyme IIB component of PTS	18.02	7.99	76
1786382	AccA acetyl CoA carboxylase carboxytransferase alpha subunit	17.83	9.36	221
2367277	YifE conserved protein UPF0438 family	13.06	13.47	196
87082236	MreB cell wall structural complex MreBCD actin like component MreB	8.01	2.14	NA[c]
1790842	DeoA thymidine phosphorylase	7.23	5.03	NA[c]
1789102	TruD tRNA Glu pseudouridine 13 synthase	5.14	3.47	NA[c]

[a] Mean protein quantities and standard deviations were calculated from at least two independent biological replicates

[b] Abundance ranking was calculated from[34]

[c] Abundance was not determined in[3]

Supplementary Table 4: Biochemical characteristics of proteins tested for HOCl-mediated chaperone activity

Protein	MW	localization[a]	chaperone activity	Cys count	% Cys	disulfide bonds[a]	Met count	% Met	Lys count	% Lys	Arg count	% Arg	GRAVY[b]	pI
RidA	13.6	cytoplasmic	yes (high)	1	0.8	0	1	1.8	8	6.2	5	3.9	0.086	5.36
BSA	66.3	extracellular	yes (high)	35	6.0	17	4	0.7	59	10.1	23	4.0	-0.469	5.65
β-lactoglobulin	18.3	extracellular	yes (med)	5	3.1	2	4	2.5	15	9.3	3	1.9	-0.162	4.83
DUK114	14.9	cytoplasmic	yes (med)	3	2.2	NA	3	2.2	13	9.4	4	2.9	0.041	7.64
α-amylase	55.4	extracellular	yes (med)	12	2.4	5	9	1.8	19	3.8	28	5.6	-0.369	6.35
RNaseA	13.7	extracellular	no	8	6.5	4	4	3.2	10	8.1	4	3.2	-0.663	8.64
Lipase	50.0	extracellular	no	14	3.1	6/7	4	0.9	42	4.9	30	4.7	-0.356	5.73
Lysozyme	14.3	extracellular	aggregates	8	6.2	4	2	1.6	6	4.7	11	8.5	-0.472	9.32

[a]according to UniProt[4]
[b]grand average of hydropathicity (GRAVY) according to Protein Identification and Analysis Tools on the ExPASy Server[5]

SUPPLEMENTARY REFERENCES

1. Datsenko, K. A. & Wanner, B. L. One-step inactivation of chromosomal genes in *Escherichia coli* K-12 using PCR products. *Proc. Natl. Acad. Sci. U.S.A.* **97,** 6640–6645 (2000).
2. Lindemann, C. *et al.* Redox proteomics uncovers peroxynitrite-sensitive proteins that help *Escherichia coli* to overcome nitrosative stress. *J Biol Chem* **288,** 19698–19714 (2013).
3. Lu, P., Vogel, C., Wang, R., Yao, X. & Marcotte, E. M. Absolute protein expression profiling estimates the relative contributions of transcriptional and translational regulation. *Nat. Biotechnol.* **25,** 117–124 (2007).
4. The UniProt Consortium. Activities at the Universal Protein Resource (UniProt). *Nucleic Acids Res.* **42,** 7486–7486 (2014).
5. Gasteiger E., Hoogland C., Gattiker A., Duvaud S., Wilkins M.R., Appel R.D., Bairoch A.; Protein Identification and Analysis Tools on the ExPASy Server*;* (In) John M. Walker (ed): The Proteomics Protocols Handbook, Humana Press (2005). pp. 571-607
